# A longitudinal cohort study to explore the relationship between depression, anxiety and academic performance among Emirati university students

**DOI:** 10.1186/s12888-020-02854-z

**Published:** 2020-09-11

**Authors:** Suheir Awadalla, E. Bethan Davies, Cris Glazebrook

**Affiliations:** 1 Division of Psychiatry and Applied Psychology, University of Nottingham, Nottingham, NG7 2UH UK; 2 College of Natural and Health Sciences Department of Psychology, Zayed University, Dubai, United Arab Emirates; 3 NIHR MindTech MedTech Cooperative Institute of Mental Health, University of Nottingham Innovation Park, Jubilee Campus, Triumph Road, Nottingham, NG7 2TU UK

**Keywords:** Depression, Anxiety, Academic performance, University students

## Abstract

**Background:**

Many university students experience depression and anxiety, both of which have been shown to affect cognitive function. However, the impact of these emotional difficulties on academic performance is unclear. This study aims to determine the prevalence of depression and anxiety in university students in United Arab Emirates (UAE). It further seeks to explore the relationship between emotional difficulties and students’ academic performance.

**Methods:**

This longitudinal study recruited 404 students (aged 17–25 years) attending one UAE university (80.4% response rate). At baseline, participants completed a paper-based survey to assess socio-economic factors and academic performance, including most recent grade point average (GPA) and attendance warnings. PHQ-9 and GAD-7 scales were used to assess depressive and anxiety symptoms. At six-month follow-up, 134 participants (33.3%) provided details of their current GPA.

**Results:**

Over a third of students (34.2%; CIs 29.7–38.9%) screened for possible major depressive disorder (MDD; PHQ-9 ≥ 10) but less than a quarter (22.3%; CIs 18.2–26.3%) screened for possible generalized anxiety disorder (GAD; GAD-7 ≥ 10). The Possible MDD group had lower GPAs (*p* = 0.003) at baseline and were less satisfied with their studies (*p* = 0.015). The MDD group also had lower GPAs at follow-up (*p* = 0.035). The Possible GAD group had lower GPAs at baseline (*p* = 0.003) but did not differ at follow-up. The relationship between GAD group and GPA was moderated by gender with female students in the Possible GAD group having lower GPAs (*p* < 0.001) than females in the Non-GAD group. Male students in the Possible GAD group had non-significantly higher GPA scores. Higher levels of both depression and anxiety symptoms scores were associated with lower GPAs at baseline. PHQ-9 scores, but not GAD-7 scores, independently predicted lower GPA scores at follow-up (*p* = 0.006). This relationship was no longer statistically significant after controlling for baseline GPA (*p* < 0.09).

**Conclusion:**

This study confirms previous findings that around a third of university students are likely to be experiencing a depressive disorder at any one time. Furthermore, it provides important evidence regarding the negative impacts of emotional difficulties on students’ academic performance. The results support the need to consider the mental health of students who are struggling academically and highlight the importance of signposting those students to appropriate support, including evidence-based therapies.

## Background

University students represent a group of people who are typically experiencing a critical transition period from adolescence to adulthood: a time often considered as one of the most stressful in a person’s life [1]. This, combined with other challenges such as social changes and exam pressures, arguably puts university students at particular risk in terms of mental health. It has been estimated that around a third of students are likely to be experiencing moderate to severe depression at any one point in time [2, 3], a rate that may exceed that found in the general population [4]. Students with fewer socioeconomic resources appear to be particularly vulnerable [5]. According to Beck’s cognitive theory of depression [6], depressed individuals in achievement-oriented environments (such as higher education institutions) are likely to react to low grades with a sense of failure and low self-esteem because of their tendency to negative cognitions of themselves, the world, and the future. Moreover, students who have a negative view of themselves may be reluctant to engage in challenging academic assignments, thereby negatively affecting their academic potential [7].

Depression is characterized by a combination of physical, emotional, psychomotor and cognitive impairments which can manifest itself by symptoms such as sleep disturbance, poor concentration, negative thoughts and feelings of guilt [8]. However, despite the marked rates of depression in student populations and the obvious potential negative implications for academic study, surprisingly little research has explored the impact of depression on academic performance. One of the few longitudinal studies to explore the relationship between emotional difficulties and objective academic performance found that pre-university depression and financial difficulties were related to exam performance at the end of the first year of study, with depression being the only independent predictor [9]. Hysenbegasi and colleagues [10] found that students with a history of depression had poorer academic performance, but that this association disappeared if their depression had been treated. Apart from acting as a barrier to help-seeking, depression can have a negative impact on engagement with their course of study. Students experiencing depression may miss more classes, tests and assignments, are more likely to drop courses compared to their non-depressed peers [11] and are more likely to dropout from university entirely [12]. Students experiencing depressive symptoms may be caught in a vicious cycle in which depression disrupts academic study and poor academic performance contributes to low mood [13]. On the other hand, a recent study carried out by Ngasa and colleagues [14] among medical undergraduate students suggested that, in spite of the high prevalence of major depression among the students, it had no effect on their self-reported GPA.

The relationship between anxiety and academic performance is more complex. Some cross-sectional studies have found that more anxious students have poorer performance [15]. However, that association may reflect students’ concerns about their studies since longitudinal studies have not shown a predictive link between high anxiety and subsequent poorer academic performance [9]. Indeed, some studies have suggested that higher levels of anxiety may be associated with better performance [16]. Therefore, this study does not propose a hypothesis in relation to the association between anxiety and academic performance.

Stigma related to depression can hinder a person’s ability to engage in university life and social activities as well as impacting on academic performance. For example, a recent systematic review of 34 studies exploring stigma associated with mental illness and its treatment in Arab culture [17] highlighted a number of ways in which a range of widely reported negative beliefs could impact on access to mental health care. This included concerns about the use of medication and negative attitudes to people with mental health conditions and to mental health professionals. Religion was found to have a strong influence on beliefs with mental illness often viewed as a curse or punishment. Despite evidence that depression and anxiety form a significant mental health problem for university students, the mental health of students in Middle Eastern countries has received little attention from researchers [18]. A better understanding of the prevalence of mood disorders in Emirati student populations and their implications for academic outcomes could help to address this gap.

### Objectives

1. To determine the prevalence of anxiety and depression in university students in United Arab Emirates (UAE).
2. To explore the relationship between anxiety or depression and academic performance (GPA) in university students in UAE.

It is hypothesized that students with a possible depressive disorder will have lower grade point averages (GPA) and that higher levels of depressive symptoms at baseline will predict lower GPAs at follow-up.

## Methods

### Participants

Participants were recruited from two campuses of one governmental university in UAE (during 2018/19 academic year). Cluster random sampling was used to recruit participants from all six faculties of the university: Business, Education, Health and Natural Sciences, Information Technology, Art & Design, and Faculty of Communication. The number of the students recruited from each faculty was determined according to its proportion of the total number of students in the university. Stratified random sampling was used to select which classes in each faculty would be asked to complete the questionnaire. Based on an estimated prevalence of depression among university students of 33.0% [2], a total university population of 9585 and a conservative response rate of 65%, it was calculated [19] that the study needed to approach 500 students in order to achieve a minimum sample size of 384 students. This allowed estimation of prevalence of depression with 5% precision and 95% confidence. Participants in their first year of study were not eligible to participate as they had not yet taken end of semester exams, and so did not have a GPA score.

### Design

The study design consists of a longitudinal survey conducted at baseline and at 6 months follow-up, with a representative cohort of students attending one university in UAE.

### Measures

#### Demographic and socio-economic factors

Information was collected about participants’ age, sex, marital status, major, year of study and parental educational level. The Family Affluence Scale (FAS) was used to assess familial material resources. The FAS comprises four items assessing parental car ownership, sharing or not sharing bedroom in the family home, computer ownership at home, and number of family holidays per year [20]. Scores range from 0 to 9 with higher scores indicating greater affluence. The FAS is a validated measure of socio-economic status and lower scores have been associated with higher risk of depression in university students [5].

#### Depressive symptoms

The Patient Health Questionnaire (PHQ-9) [21] is a short screening measure used in clinical and community settings to assess symptoms of depression. It consists of nine items that correspond to the nine DSM-IV criteria for depression (DSM–IV) [22]. Each item is rated for the previous 2 weeks prior to administration: each item is scored on a scale from zero (‘not at all’) to three (‘nearly every day’), with a total score ranging from 0 to 27. In this study, participants scoring ≥10 were allocated to the Possible major depressive disorder (MDD) group and students scoring ≤9 were allocated to the Non-MDD group [21]. Although the PHQ-9 is not a diagnostic tool, a cut-off score of 10 for PHQ-9 has been shown to have a sensitivity of 88% and a specificity of 88% for the classification of MDD [23].

#### Anxiety symptoms

The General Anxiety Disorder 7-item scale (GAD-7) is a short screening tool used to measure the severity of generalized anxiety disorder (GAD). It is a self-report scale consisting of seven items based on DSM-IV criteria and has excellent internal consistency (α = .89–.92) [24]. Spitzer and colleagues [25] proposed that it is a useful screening tool with strong criterion validity for identifying probable cases of GAD. In addition, the GAD-7 has been strongly associated with multiple domains of functional impairment and cognitive disability [25]. The GAD-7 total score ranges from 0 to 21. In this study participants scoring 10 or above were allocated to the Possible GAD group and students with GAD-7 scores below 10 were allocated to the Non-GAD group [25]. A cutoff score of 10 for GAD-7 was classified as the optimal point for sensitivity 89% and specificity 82% for screening for possible GAD [25].

### Academic performance

Participants provided information about their most-recent grade point average (GPA), number of academic warnings about poor attendance in the last semester, and level of satisfaction with their course of study. Possible GPA scores range from 0 to 4, with higher scores indicating better performance. Satisfaction with studies was ranked on a 0 to 6 scale from ‘extremely satisfied’ to ‘extremely dissatisfied’, with lower scores indicating greater satisfaction.

### Ethical approval

Ethical approval was obtained from the Division of Psychiatry and Applied Psychology Ethics Sub-Committee (reference number: 250) and the Research Ethics Committee at Zayed University (ref ZU17_0107 F). Participation was voluntary and confidentiality was assured. Informed written consent to participate was obtained.

### Procedure

An invitation email and participant information sheet explaining the study were sent to all students in the selected classes in each faculty by academic staff in the week before data collection. An anonymous paper questionnaire comprising demographic items, PHQ-9, GAD-7 and academic performance items was distributed to students in the pre-selected classes by the researcher in March 2018. Participation was entirely voluntary, and students consented by ticking the paper consent form attached to the anonymous baseline questionnaire. In order to link the questionnaire responses at baseline with academic performance at follow-up, participants generated their own personal study number by providing their day of birth and the last three digits of their phone number. Students completed the academic performance questionnaire again at the beginning of the next semester (approximately 6 months later).

### Statistical analysis

Data were analysed using SPSS v.24 (SPSS, 2017). Non-parametric correlations were performed to explore the relationship between socioeconomic factors and anxiety and depression and to explore factors associated with poor academic performance. Chi-square was used to assess associations for nominal data and group differences in ordinal and continuous data were assessed using Mann-Whitney U tests or t-tests as appropriate. Linear regressions (entry method) were used to explore independent predictors of poor academic performance. Two-way ANOVAs were used to explore the possible moderating effect of gender on the relationship between MDD or GAD group and GPA.

## Results

A total of 404 of 500 undergraduate students (300 females and 200 males) were recruited to the study (80.8% response rate). The response rate for the female students was significantly higher than for male students (96.7% vs 57.5%; X^2^ = 123.37, df = 1, *p* < 0.001). No student refused to participate, but 96 students (19.2%) were absent from class when the baseline questionnaires were distributed. All students recruited to the study (baseline responders) completed the baseline questionnaire. The sample’s mean age was 19.6 (SD = 2.76) years (range 17–25), and the sample was almost three-quarters female (*n* = 290, 72.2%). There was a broad distribution in the educational level of participants’ parents, with 56.7% (*n* = 229) of paternal and 48.5% (*n* = 196) of maternal being university graduates. The majority of participants were single (*n* = 381, 94.3%). Table 1 shows the demographic make-up of the sample. According to the results of this study at baseline males are older than females, come from more affluent families and have less educated parents than females.

**Table 1:** Demographic composition of the sample at baseline (*N* = 404) and follow-up (*N* = 138)

Variables	Baseline (***N*** = 404) n (%)	Follow-up (***n*** = 138) n (%)
**Gender**		
Male	114 (28.2%)	41 (30.6%)
Female	290 (72.2%)	93 (69.4%)
**Age**		
17–20	292 (72.3%)	94 (71.2%)
21–25	91 (22.5%)	33 (25%)
26–30	8 (1.9%)	4 (3%)
31–41	4 (0.8%)	1 (.8%)
Mean age (SD)	19.64 (2.75)	19.8(2.91)
**Marital status**		
Married	20 (5.0%)	6 (4.5%)
Divorced	2 (.5%)	0
Single	381 (94.3%)	128 (95.5%)
**Year of study**		
2nd year	303 (75.0%)	95 (72%)
3rd year	65 (16.1%)	23 (17.4%)
4rd year	30(7.4%)	14 (10.6%)
**Faculty of study**		
Business	138 (34.0%)	49 (36.6%)
Humanities & Social Sciences	77 (19.1%)	21 (15.7%)
Technological Innovation	71 (17.6%)	23 (17.2%)
Communication & Medical Sciences	57 (14.1%)	25 (18.7%)
Natural Health Sciences	46 (11.4%)	16 (11.9%)
**Maternal level of education**		
None/below secondary school education	43 (10.6%)	12 (9%)
Completed secondary school education	165 (40.8%)	52 (38.8%)
Completed college university education	196 (48.5%)	70 (52.2%)
**Paternal level of education**		
None/below secondary school education	41 (10.0%)	14 (10.4%)
Completed secondary school education	131 (32.4%)	41 (30.6%)
Completed college university education	229 (56.7%)	79 (59%)
**Family Affluence Scale**		
Mean (SD)	7.09 (1.52)	7.01 (1.57)
Median (range)	7.00 (2–9)	7.02(2–7)

### Prevalence of depression and anxiety in the sample and socioeconomic factors associated with poorer mental health

At baseline, 34.2% (*n* = 138, CIs 29.7–38.9%) of students scored above the cut-off for possible MDD (Table 2). Females had higher PHQ-9 scores compared to males (Z = 3.63, *p* < 0.001,) although rates of possible MDD were similar between males (31.6%) and females (35.3%). Students with higher PHQ-9 scores came from less affluent families (r_s_ = −.276, *n* = 404, *p* < 0.001) and had less maternal education (r_s_ = −.118, *n* = 403, *p* < 0.05). PHQ-9 scores were unrelated to year of study, participant age or paternal educational level (all *p*= > .05).

**Table 2:** Mental health and academic outcomes at baseline (*n* = 404)

Scale	Males (*n* = 114)	Females (*n* = 290)	Total (*N* = 404)
Mean PHQ-9 score (SD)	6.29 (5.65)	8.29 (5.03) ***	7.76 (5.29)
Possible MDD group	36 (31.6%)	102 (35.3%)	138 (34.2%)
Mean GAD-7 score (SD)	4.22 (4.57)	6.75 (5.13) ***	6.07 (5.12)
Possible GAD group	16(14.3%)	73 (25.2%) *	89(22.3%)
GPA	2.73 (0.60)	2.83 (0.67)	2.8 (0.65)
Attendance warning Median (range)	1.00(0–3)	0.00(0–6)	0.00(0–6)
Study satisfaction Mean (SD)	3.90(1.65)	3.54(1.74)	3.64(1.74)

*p = < 0.05, *p* = < 0.001 ***

Eighty-nine students (22.3%, CIs 18.2–26.3%) scored above the cut-off for possible GAD (Table 2). Total GAD-7 anxiety scores were higher in females (Z = -4.77, *P* < 0.001,*)* and female students were also more likely to be in the possible GAD group compared to males (X^2^ = 6.063, df = 1, *P* = 0.014). Students with higher levels of anxiety symptoms came from less affluent families (r_s_ = −.271, *n* = 401, *p* < 0.001) and had less maternal education (r_s=_ − .167, *n* = 404, *p* < 0.01). Levels of anxiety were unrelated to year of study, participant age or paternal level of education. PHQ-9 scores were positively and strongly correlated with GAD-7 scores (r_s_ = .736, *n* = 404, *p* < 0.001).

Students’ GPA at baseline ranged from 0.66 to 4.00 with a mean of 2.8 (0.65): *n* = 48 (11.9%) students had a GPA below 2.00 indicating poor academic performance and triggering academic support within the university. Students were moderately satisfied with their studies, with a median score of 4 out of 7 with lower scores indicating greater satisfaction. Nearly half of the sample had received at least one attendance warning (*n* = 171, 47.8%). Students with lower GPAs had had more attendance warnings (r_s_ = − 0.24, *n* = 376, *p* < 0.01) and were less satisfied with their studies (*r* = − 0.182, *n* = 397, *p* < 0.01) (Table 3). Poorer academic performance was also associated with older age (*r* = −.176 *n* = 398 *p* = 0.01), higher year of study (*r* = − 0.17, *n* = 392, p < 0.01), lower level of paternal education (*r* = .127, *n* = 395, *p* = .011), and lower level of maternal education (*r* = .11, *n* = 398, *p* = .028). There was no relationship between GPA and family affluence or gender.

**Table 3:** Possible MDD and academic outcomes at baseline and follow-up

	Baseline (*N* = 404)		Follow-up (*N* = 138)	
	Possible MDD (*N* = 136) Mean (SD)	Non MDD(*N* = 261) Mean (SD)	Possible MDD (*N* = 58) Mean (SD)	Non MDD(*N* = 76) Mean (SD)
Semester GPA	2.67 (0.70)	2.87 (0.61) **	2.40 (0.70)	2.68 (0.76) **
Attendance warnings	0.93 (1.05)	0.77 (1.07)	1.12 (1.03)	0.92 (1.00)
Satisfaction level	3.78 (1.58)	3.60 (1.85) *	3.98 (1.42)	3.71 (1.55)

Means and SD at baseline and six-month follow-up

*p = < 0.05, **p = < 0.01

### Relationship between depressive symptoms and academic performance at baseline

At baseline, students with higher PHQ-9 scores had lower current GPAs (r_s_ = −.171, *n* = 397, *p* < 0.001). There was no relationship between PHQ-9 scores and number of attendance warnings or satisfaction with course. As hypothesized, students in the Possible MDD group (*n* = 136) had lower GPA scores compared to students below the cut-off (t = 2.98 *p* = 0.003, *d* = 0.3). They were also less satisfied with their studies (Z = 2.42, *p* = 0.015) but there was no relationship with number of attendance warnings (Table 3).

Regression analysis (entry method) with baseline GPA as the dependent variable and PHQ-9 scores, GAD-7 scores, age, gender and maternal education as independent variables found that, although the model was significant (F = 5.617, df = 3388, *p* < 0.001), it accounted for only a small proportion of variance (R^2^ = 0.056). Only age was significantly related to GPA, with older students having poorer GPA (B = −.034, t = − 2.59, *p* = 0.01). However, although there was no evidence of serious problems with multicollinearity, GAD-7 scores were just above the threshold for an acceptable variance inflation factor (VR = 2.06) (Table 4).

**Table 4:** Regression analysis to explore relationship between depression, anxiety and baseline GPA

Predictors	Baseline GPA			
	*B*	*SE B*	*β*	*T*
Age	−.034	.013	−.145*	−2.595**
Gender	.088	.082	.062	1.085
Maternal level of education	.064	.051	.066	1.266
PHQ-9 scores	−.011	.009	−.088	−1.276
GAD-7 scores	−.014	.009	−.111	−1.584

GPA at baseline as dependent variable

*p = < 0.05, **p = < 0.01

A two-way ANOVA with gender and MDD group as the independent factors, GPA as the dependent variable and age as the covariant, found a main effect of MDD group (F = 6.692, df = 1389, *p* = 0.01) with Possible MDD group having higher scores but no gender effect and no interaction between gender and MDD group.

### Relationship between anxiety and academic performance at baseline

Higher GAD-7 scores (indicating more symptoms of anxiety) were associated with poorer GPA scores (r_s_ = −.176, *n* = 398, *p* > 0.001). However, there was no independent relationship between GAD-7 scores and baseline GPA once depression and sociodemographic variables had been accounted for (Table 4). There was no relationship between total GAD-7 scores and number of attendance warnings or satisfaction with studies rating. Students above the cut-off for possible GAD had lower GPA scores (mean 2.62, SD = 0.71) in comparison to students below the cut-off (mean 2.86, SD = 0.62) (t = 3.04 *p* = 0.003 *d* = 0.4) but did not differ in terms of number of attendance warnings or rating of satisfaction with course (Table 5).

**Table 5:** GAD group and academic outcomes at baseline and follow-up

	Baseline (*N* = 404)		Follow-up (*N* = 138)	
	Possible GAD (*N* = 87) Mean (SD)	Non-GAD(*N* = 309) Mean (SD)	Possible GAD (*N* = 43) Mean (SD)	Non-GAD(*N* = 91) Mean (SD)
Semester GPA	2.62 (0.71)	2.86 (0.62) **	2.43 (0.70)	2.62 (0.76)
Attendance warnings	1.02 (1.16)	0.75 (1.01)	1.19 (1.12)	0.92 (0.96)
Satisfaction level	3.77 (1.59)	3.63 (1.80)	3.95 (1.45)	3.77 (1.48)

Means and SD at baseline and six-month follow-up

**p = < 0.01

Two-way ANOVA with GAD group and gender as independent factors, age as covariate and baseline as dependent variable, found no main effects of group or gender, but a significant group by gender interaction (F = 5.75, df = 1387, eta =0.15). The effect of GAD group on GPA was moderated by gender: females in the Possible GAD group had lower GPA (mean 2.91 SD, 0.62) than those in Non-GAD group (mean 2.57, SD 0.73) (t = 3.8, df = 280, *p* < 0.001 d = 0.48). In contrast, males in the Possible GAD group had non-significantly higher GPA (mean 2.71 SD 0.61) compared to males in the Non-GAD group (2.82 SD 0.59).

### Factors influencing GPA at follow-up

A total of 134 (93 females and 41 males) completed the follow-up survey (follow-up responders), a 33.3% follow-up response rate. Follow-up responders were significantly more likely to be in the Possible MDD group (43.0% vs 29.0%; X^2^ (1) = 11.54, p = < 0.01) at baseline and to have higher PHQ-9 scores (8.84, SD = 5.25 vs mean 7.20 SD = 5.25; Z = -3.37 *p* < 0.01) compared to non-responders at follow-up. Similarly, follow-up responders were more likely to be in the Possible GAD group (32% vs 17%; X^2^ (1) =7.29, p < 0.01) at baseline and to have higher GAD-7 scores (2.62, SD 0.71 vs 2.43, SD 0.70; *p* < 0.05). There were no significant differences in baseline GPA, satisfaction with studies or number of attendance warning between responders at follow-up and non-responders at follow-up.

### Relationship between levels of anxiety and depression at baseline and academic performance at follow-up

Higher PHQ-9 scores at baseline were associated with lower GPA scores at follow-up (r_s_ = − 0.24, *n* = 134, *p* = 0.006), and students in the Possible MDD group (*n* = 58) had significantly lower GPA scores at follow-up compared to students in the Non-MDD group (*n* = 76) (mean 2.40 vs 2.68, t = 2.13, *p* = 0.035, *d* = 0.4) (Table 3). There was no relationship between GAD-7 scores and follow-up GPA and no significant difference in GPA at follow-up between participants in the Possible GAD group and the Non-GAD group. Higher GPA scores at follow-up were associated with younger age (r_s_ = −.0162, *n* = 134, *p* = 0.05) but there was no relationship between family affluence, parental education or gender and follow-up GPA.

Regression analysis (entry method) was conducted with GPA at follow-up as the dependent variable and PHQ-9 scores, GAD-7 scores, age, and gender as independent variables.

The overall model was significant (F = 2.66, df = 4127, *p* = 0.035) but only accounted for a small proportion of variance (adjusted R^2^ = 0.048). Only PHQ-9 scores (B = −.043, *p* = 0.014) at baseline predicted GPA at 6 months follow-up (Table 6).

**Table 6:** Regression analysis to explore relationship between PHQ-9 scores at baseline and GPA at follow-up

Predictors	Follow-up GPA			
	*B*	*SEB*	β	*T*
(Constant)	2.612	.524		4.314
PHQ-9 scores	-.043	.017	−.302	−2.500**
GAD-7 scores	.011	.017	.083	.682
Age	.022	.024	.085	.914
Gender	.248	.151	.153	1.640

Semester GPA at follow-up as dependent variable

**p = < 0.01

Including baseline GPA into the regression meant that the relationship between depressive symptoms at baseline and follow-up GPA just failed to reach significance (B = -.019, *p* = 0.09) and the total adjusted r^2^ increased to 0.633 reflecting the fact that baseline GPA accounted for a high proportion of the variance in GPA at follow-up.

## Discussion

This representative survey found just over a third of students (34.2%) scored over the threshold for possible Major Depressive Disorder on the PHQ-9. Although female students had higher PHQ scores they did not differ statistically from males in terms of rates of MDD. A smaller proportion of students (22.3%) scored above the cut-off for possible generalized anxiety disorder, with females having higher rates of GAD-7 scores. Cross-sectional analyses found that higher levels of both depression and anxiety were significantly but weakly associated with poorer academic performance. Students scoring above the cut-offs either for possible MDD or possible GAD had lower GPA scores, although the effect sizes were small. There was evidence that gender moderated the relationship between GPA scores and anxiety as females in the Possible GAD group had substantially poorer GPA scores compared to females in the Non-GAD group, but there was no evidence that Possible GAD group influenced GPA in males. Longitudinal analysis found depression, but not anxiety, predicted poorer GPA at six-months follow-up. However, this just failed to reach significance (p = < 0.09) after controlling for baseline GPA.

In the present sample, the level of depression appears high compared to other student-sampled studies in similar communities, but this probably reflects differences in the classification of depression. For example, a study carried out at Al Ain University in UAE found a prevalence rate for depression of 22.2% [8], but although this study used the PHQ-9 it used the higher cut-off of 11 and required students to endorse specific items in order to be classified as having possible MDD. Another study in Oman [26] estimated a prevalence of 27.7% also using a cut point of 11. That study recruited through the university health clinic which may also have impacted on the reported prevalence. The prevalence in the present sample (34.2%; CIs 29.7–38.9%) is similar to that found in a systematic review of 24 studies of depression among university students [3] which estimated a weighted mean prevalence of 30.6% (CIs 95% CI, 30.2–31.1) in student populations.

Consistent with previous studies [10, 27, 28], baseline findings support an association between higher levels of depressive symptoms and poorer academic performance among university students. However, this relationship was no longer significant once other factors such as anxiety and age had been controlled for. The results signified that students scoring above the cut-off for possible MDD (PHQ-9 score ≥ 10) have poorer academic performance. This is consistent with studies carried out by Bostani and colleagues [29] in 2014 and Eisenberg and colleagues [30] in 2009. Follow-up was conducted by the end of the semester (i.e. 6 months from baseline). Students classified as having scores above the cut-off for possible MDD had lower GPA scores at follow-up compared to students below the cut-off. The relationship between PHQ-9 scores at baseline and GPA at follow-up was stronger than the cross-sectional relationship, and was also independent of anxiety and sociodemographic variables, supporting a causal relationship between higher levels of depressive symptoms and poorer academic performance. These findings align with a previous study at a UK university [9] which found depression (but not anxiety) measured midway through the second year was negatively related to exam scores at the end of the second year. In a related context, Hysenbegasi and colleagues [10] in 2005 compared the GPA of 121 students over six-months following a diagnosis of depression and found a significant negative relationship between academic performance and untreated symptoms of depression. Students who received treatment for depression during the six-month period, had GPA scores that were statistically similar to the non-depressed group at follow-up, supporting a causal relationship between depression and academic performance.

Depression could impact on academic performance in a number of ways. Young people with depression may find social interactions difficult and may fail to engage with their courses [31]. However, in this study depression was not associated with poorer attendance in contrast to a recent study [32] conducted in Jordan suggesting a negative relationship between depression and high absence among nursing university students. The relationship in this study may reflect the fact that attendance is mandatory and missing classes is penalized. Whilst encouraging students to attend may help to mitigate the impact of depression, the low level of absenteeism may contribute to the difficulty of identifying academically struggling students.

The prevalence of anxiety found in the present study (22.3%) was similar to a study carried out among Malaysian student population [33], where the rate of prevalence was 27.4% taking to consideration the study used the cut-off of 8 and the current study used the cut-off of 10. Furthermore, the prevalence of anxiety in this sample was lower compared to other studies: e.g. 64.3% in Egyptian students [34] using the short-form Depression Anxiety Stress Scales and 47.1% in Turkish students [35] using the long-form Depression Anxiety Stress Scales.

Consistent with previous studies [36, 37], findings from baseline data supported a negative relationship between symptoms of anxiety and academic performance among university students. Moreover, students meeting the criteria for possible generalized anxiety disorder (GAD) had lower GPA scores compared to students in the Non-GAD group. This is consistent with studies carried out by Bostani and colleagues [29] in 2014 and Eisenberg and colleagues, [30] in 2009. In contrast, some studies found that students with moderate levels of anxiety had better academic achievement [9, 16, 38] reflecting that appropriate degrees of anxiety concerning fear of failure could enhance self-motivation of students to perform better in different academic tasks. In this study anxiety was no longer associated with GPA at baseline once other variables such as depression had been controlled for. The lack of a longitudinal relationship between anxiety and GPA has been observed in previous studies [9] and suggests that anxiety may be reflective of academic difficulties rather than causal. One possible interpretation of the interaction between gender, Possible GAD group and GPA at baseline is that for female’s poor performance contributes to anxiety but not for males. This is a novel finding and needs further investigation.

Furthermore, it should be considered that in the Middle East area, accessing help for mental health services is challenging [39]. Stigma and shortage of clinicians who are efficient and able to provide intervention that respects and integrates patients’ cultural values [40] in the health care system are the critical barriers to effective treatment [41].

Identification of low mood in students who are struggling academically and offering appropriate support may help to improve both mental health and academic outcomes but the findings from this study suggest that may be difficult as students with low mood had not had poorer attendance. Increasing mental health literacy in students and educators could reduce barriers to treatment. Academic staff are often the first to observe behaviours that indicate either the development or worsening of mental health problems among students [42]. An Australian survey found that academic staff with higher levels of depression literacy were more likely to initiate engagement with students with mental health problems and were more likely to be approached by students who wanted to discuss their mental health. Staff with higher depression literacy also felt more confident that they had the knowledge to help students with their mental health problems [43]. Staff would therefore be in a good position to signpost academically struggling students with mental health problems to sources of support and treatment.

Theory driven, web-based intervention programs offer an acceptable and effective method of providing psychological treatment within the university system. Online therapy may appeal to students who do not attend or cannot access established mental health clinics due to a range of different barriers including the stigma related to mental health issues [44]. Furthermore, web-based interventions could be combined with face-to-face support to achieve best improvements in emotional wellbeing among university students [45].

## Strengths and limitations

This appears to be first study to explore the impact of depression and anxiety on the academic performance of university students in the UAE, with a representative sample of the targeted population. In addition, the longitudinal nature of the present study assessed academic performance of the students over time to enable some conclusions about the causal nature of the relationship between emotional difficulties and subsequent academic difficulties. Furthermore, the two scales that have been used in this study for anxiety and depression (GAD-7 and PHQ-9) are validated and have excellent sensitivity and specificity. Another strength of this study was that participants lost to follow-up were similar to responders at follow-up in terms of socio-demographic and educational characteristics. However, responders at follow-up did have higher rates of baseline possible MDD and possible GAD, but the repeated measures design means that this is unlikely to have biased the findings. Some limitations were encountered in the present study. For example, the response rate at follow-up was low, which limited the potential of the study to conduct structural equation modelling to look for potential mediators for change. The survey was kept anonymous in order to maximize the baseline response rate and to ensure the veracity of students’ responses. However, this meant that it was not possible to target non-responders which contributed to the low follow-up response rate.

Another limitation was that, in order to preserve anonymity, we had to rely on self-reported GPA for measuring academic performance. Finally, the sample of this study consists of a group of students in just one university in the UAE, limiting the generalizability of the results.

## Conclusion

Findings in the current study indicated that higher levels of depressive and anxiety symptoms affected around a third of university students in the sample. Cross-sectional analyses show that students scoring above the cut-off for possible MDD or possible GAD have poorer academic performance. Depressive symptoms predict GPA in the subsequent semester suggesting a causal link but as students with possible MDD did not have reliably poorer attendance it may be difficult to identify at-risk students. This study provides evidence regarding the negative impacts of emotional difficulties on students’ academic achievements and chances of success, accordingly academic counsellors should consider routinely signposting academically failing students to mental health support. Future research could distinguish the impacts of different types of anxiety disorder, particularly social anxiety which is common in university students and may impact on non-exam-based assessments such oral presentations [46].

## Data Availability

The datasets generated during and/or analysed during the current study are not publicly available [PhD study under progress] but are available from the corresponding author on reasonable request.

## Abbreviations

GPA: Grade point average
MDD: Major depressive disorder
GAD: General anxiety disorder
UAE: United Arab Emirates
PHQ-9: Patient Health Questionnaire (9-item version)
GAD-7: General Anxiety Disorder scale (7-item version)
SDs: Standard deviation
FAS: Family affluence scale
CI: Confidence interval
ANOVA: Analysis of variance
